# AVR2 Targets BSL Family Members, Which Act as Susceptibility Factors to Suppress Host Immunity^1^

**DOI:** 10.1104/pp.18.01143

**Published:** 2019-02-19

**Authors:** Dionne Turnbull, Haixia Wang, Susan Breen, Marek Malec, Shaista Naqvi, Lina Yang, Lydia Welsh, Piers Hemsley, Tian Zhendong, Frederic Brunner, Eleanor M. Gilroy, Paul R.J. Birch

**Affiliations:** a Division of Plant Science, School of Life Science, University of Dundee (at JHI), Invergowrie, Dundee DD2 5DA, United Kingdom; b Key Laboratory of Horticultural Plant Biology (HZAU), Ministry of Education, Key Laboratory of Potato Biology and Biotechnology, Ministry of Agriculture, Huazhong Agricultural University, Wuhan, Hubei 430070, China; c Cell and Molecular Science, James Hutton Institute, Invergowrie, Dundee DD2 5DA, United Kingdom; d Department of Biochemistry, Centre for Plant Molecular Biology, Eberhard Karls University, Auf der Morgenstelle 32, D-72076 Tübingen, Germany; e Fujian Key Laboratory of Plant Virology, Institute of Plant Virology, Fujian Agricultural and Forestry University, Fuzhou, Fujian 350002, China (L.Y.)

## Abstract

To be successful plant pathogens, microbes use “effector proteins” to manipulate host functions to their benefit. Identifying host targets of effector proteins and characterizing their role in the infection process allow us to better understand plant–pathogen interactions and the plant immune system. Yeast two-hybrid analysis and coimmunoprecipitation were used to demonstrate that the *Phytophthora infestans* effector AVIRULENCE 2 (PiAVR2) interacts with all three BRI1-SUPPRESSOR1-like (BSL) family members from potato (*Solanum tuberosum*). Transient expression of BSL1, BSL2, and BSL3 enhanced *P. infestans* leaf infection. BSL1 and BSL3 suppressed INFESTIN 1 elicitin-triggered cell death, showing that they negatively regulate immunity. Virus-induced gene silencing studies revealed that BSL2 and BSL3 are required for BSL1 stability and show that basal levels of immunity are increased in *BSL*-silenced plants. Immune suppression by BSL family members is dependent on the brassinosteroid-responsive host transcription factor CIB1/HBI1-like 1. The *P. infestans* effector PiAVR2 targets all three BSL family members in the crop plant *S. tuberosum*. These phosphatases, known for their role in growth-promoting brassinosteroid signaling, all support *P. infestans* virulence and thus can be regarded as susceptibility factors in late blight infection.

## Abstract (precis)

Late blight pathogen Phytophthora infestans uses effector Avr2 to target BSL phosphatases to suppress plant immunity.

Plants are constantly challenged by microbes, such as bacteria, fungi, and oomycetes, the majority of which are nonpathogenic by virtue of the highly effective plant immune system. However, those that do cause disease have the potential to devastate crop yields, with pathogens responsible for 10% to 16% loss of our global harvest (Chakraborty and Newton, 2011). Faced with the challenge of increasing food production to feed a growing population, our research seeks to understand the complexity of plant–pathogen interactions: How does a pathogen cause disease, and how does the plant recognize and respond to it? The plant immune system can be triggered in two main ways: first, by detection of broadly conserved microbe/pathogen-associated molecular patterns (MAMPs/PAMPs), which may be structural elements, such as bacterial flagellin and fungal chitin, or secreted proteins, such as the oomycete elicitin INFESTIN 1 (INF1). MAMPs/PAMPs are recognized by transmembrane proteins called pattern recognition receptors to elicit pattern-triggered immunity (PTI; Jones and Dangl, 2006). Second, specific pathogen “effector proteins” can be perceived either directly or indirectly by resistance (R) proteins, activating effector-triggered immunity. Immune activation can trigger considerable physiological change in the plant, including differential gene transcription, mitogen-activated protein kinase activation, production of reactive oxygen species, callose deposition, and a form of programmed cell death called the hypersensitive response (HR; Feechan et al., 2015). Effector-triggered immunity is also associated with systemic acquired resistance, in which pathogen recognition results in systemic signaling to prime the whole plant for defense (Durrant and Dong, 2004).

The effector protein repertoire can be viewed as a pathogen “toolkit,” a combination of secreted proteins that facilitate manipulation of the host plant to the advantage of the microbe. Effectors play a variety of roles in pathogenicity, from suppressing the plant immune response to providing physical and metabolic alterations that support infection. Immune suppression can be broadly achieved in two ways: (1) by reducing the activity of a positive immune regulator or (2) by enhancing the function of a negative immune regulator. The late blight pathogen *Phytophthora infestans* has been shown to use both strategies to achieve virulence (Whisson et al., 2016). Whereas the RXLR (Arg−any amino acid−Leu−Arg motif) effectors AVIRULENCE 3a (AVR3a), PITG_11383 (PexRD2), PITG_03192 (Pi03192), and SUPPRESSOR OF EARLY FLG22-INDUCED IMMUNE RESPONSE 3 (SFI3) reduce defenses by means of interaction with positive regulators of immunity (Bos et al., 2010; Gilroy et al., 2011b; McLellan et al., 2013; King et al., 2014; He et al., 2018a), recent research has shown that RXLR effectors Pi04089, Pi04314, Pi02860, and Pi17316 interact with negative regulators of immunity (Wang et al., 2015; Boevink et al., 2016a; Yang et al., 2016; He et al., 2018b; Murphy et al., 2018). These effector targets are capable of attenuating *P. infestans* infection when silenced, increasing *Phytophyhora infection* colonization when overexpressed, or both, classifying them as “susceptibility (S) factors” in late blight infection (van Schie and Takken, 2014; Boevink et al., 2016b; Whisson et al., 2016).

Phytohormones, such as salicylic acid, ethylene, and brassinosteroids, are intrinsic to integrating environmental cues. While initially recognized for their effects on plant growth and development, they are also perceived to play important roles in defense and immunity, such as salicylic acid-mediated resistance to biotrophic pathogens and ethylene/jasmonic acid signaling associated with resistance to necrotrophs. Hormone signaling pathways do not function in isolation, and both complementary and opposing effects have been described. The negative cross talk between growth-promoting brassinosteroid signaling and the plant immune response is well characterized. Brassinosteroid signaling begins at the plasma membrane with the perception of brassinosteroid hormone (BR) by the receptor-like kinase BR INSENSITIVE 1 (BRI1; Li and Chory, 1997). BR induces BRI1 dimerization, hetero-oligomerization, and transphosphorylation of the coreceptor BRASSINOSTEROID-ASSOCIATED KINASE 1 (BAK1; Li et al., 2002; Nam and Li, 2002), as well as phosphorylation and subsequent dissociation of the negative regulators BRI1 KINASE INHIBITOR 1 and BOTRYTIS-INDUCED KINASE 1 (Wang and Chory, 2006; Lin et al., 2013). Activated BRI1 phosphorylates the cytoplasmic tyrosine kinase CDG1 and the BSK family (Tang et al., 2008; Kim et al., 2011), which proceed to phosphorylate a family of kelch-repeat phosphatase proteins. The best characterized of these is BRI1 SUPPRESSOR1 (BSU1) in Arabidopsis (*Arabidopsis thaliana*), with other family members designated BSU1-like 1 (BSL1), BSL2, and BSL3 (Mora-García et al., 2004; Kim et al., 2009). The family is collectively referred to as the BSL family throughout this article. In addition to likely unknown substrates, the BSL family dephosphorylates and deactivates the glycogen synthase kinase BRASSINOSTEROID-INSENSITIVE2 (BIN2; Nam and Li, 2002), allowing PROTEIN PHOSPHATASE 2A (PP2A) to dephosphorylate the homologous transcription factors BRASSINAZOLE RESISTANT1 (BZR1) and bri1-EMS SUPPRESSOR1 (BES1), which can then participate in gene regulation events in the nucleus (Tang et al., 2011). BR-induced transcriptional changes include those linked to cell expansion and growth, light signaling and photomorphogenesis, and regulation of other hormone signaling pathways such as auxin and ethylene (Goda et al., 2002; Müssig et al., 2002).

The inverse correlation between growth and immune function was initially postulated to be a result of competition for the shared coreceptor BAK1, which, in addition to being required for BR signaling, is also required for immune signaling by pattern recognition receptors such as FLS2 (a protein associated with flagellin perception), EFR (LRR receptor-like serine/threonine protein kinase), and ELR (ELICITIN RESPONSE protein; Chinchilla et al., 2007; Chaparro-Garcia et al., 2011; Du et al., 2015). However, this was not the case, as BAK1 was shown not to be the rate-limiting factor between brassinosteroid and immune signaling (Albrecht et al., 2012). More recently, BR immune antagonism has been attributed to transcription factors downstream of BR perception. BZR1 activation has been shown to inhibit PTI responses, with an overrepresentation of defense-related genes under its transcriptional control (Lozano-Durán et al., 2013). BZR1 upregulates several basic helix-loop-helix (bHLH) transcription factors that act as negative regulators of immunity in Arabidopsis, such as CRYPTOCHROME-INTERACTING bHLH1 (CIB1), HOMOLOG OF BRASSINOSTEROID-ENHANCED EXPRESSION 2 INTERACTING WITH IBH1 (HBI1), and BRASSINOSTEROID-ENHANCED EXPRESSION 2. This cross talk is bidirectional; while these transcription factors are positively regulated by BR signaling, they are down-regulated by PAMP perception (Malinovsky et al., 2014).

The large, repeat-rich genome of *P. infestans* boasts more than 500 RXLR effector gene candidates (Haas et al., 2009; Vleeshouwers et al., 2011), with only a minority of these characterized to date. One of these effectors is PiAVR2, recognized by the potato (*Solanum tuberosum*) resistance protein R2 from *Solanum demissum* (Gilroy et al., 2011a). PiAVR2 interacts with the plant phosphatase StBSL1, and this interaction is a prerequisite for R2-mediated HR (Saunders et al., 2012). Our recent work has shown that PiAVR2 functions to exploit growth/immune cross talk by up-regulating brassinosteroid pathway signaling to attenuate the immune response (Turnbull et al., 2017). Markers of BR signaling were shown to be increased in PiAVR2-expressing transgenic potato, with one of these, CIB1/HBI1-like (StCHL)1, identified as a transcriptional regulator capable of suppressing innate immunity. Notably, silencing of *BSL1* has neither a developmental nor a virulence phenotype, with *P. infestans* able to infect as normal (Saunders et al., 2012). This raised the possibility of StBSL1 acting as a decoy (van der Hoorn and Kamoun, 2008), involved solely in the recognition of PiAVR2 by R2 rather than playing a role in plant development.

Data presented here show that PiAVR2 interacts not only with StBSL1, but also with the other family members, StBSL2 and StBSL3. This interaction requires a specific C-terminal region of the PiAVR2 protein, without which the effector is stripped of its virulence function and is no longer recognized by the resistance protein R2. Furthermore, all three phosphatases are capable of enhancing *P. infestans* virulence when overexpressed, identifying the StBSL proteins as susceptibility factors in late blight infection. However, this is not a straightforward case of redundancy; there are functional differences and regulatory interactions between BSL family members at both the gene expression and the protein levels. In addition, INF1 cell death suppression by StBSL1 and StBSL3 is shown to require the bHLH transcription factor StCHL1, recently shown to function downstream of BR signaling as a suppressor of immunity (Turnbull et al., 2017). This work builds upon previous functional characterization of the *P. infestans* effector PiAVR2, revealing additional complexity in its host targets, the StBSL phosphatases, and provides insight into how this oomycete effector tips the balance between growth and immunity in favor of disease.

## RESULTS

### PIAVR2 Interacts with the BSL Family in *Solanum tuberosum*

**Figure 1.**
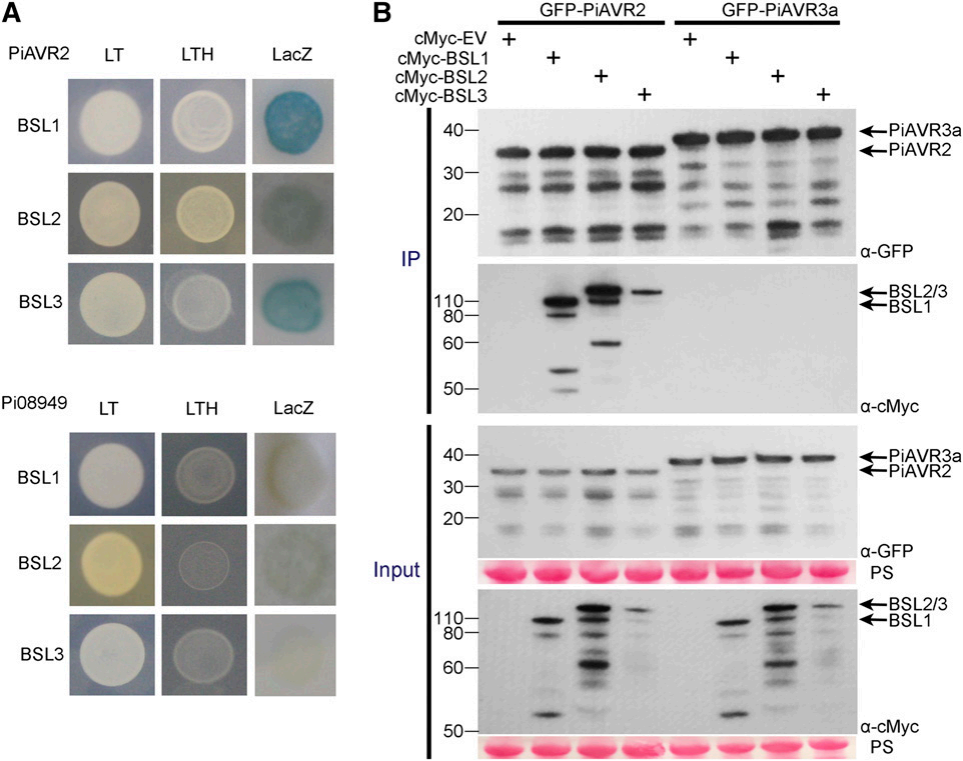
PiAVR2 interacts with StBSL1, StBSL2, and StBSL3. A, Yeast coexpressing BSL1, BSL2, or BSL3 with PiAVR2 grew on -His (LTH) medium and yielded β-galactosidase (LacZ) activity, while those coexpressed with the control effector Pi08949 did not. The +HIS (LT) control shows that all yeast were able to grow in the presence of His. B, IP of protein extracts from agroinfiltrated leaves using GFP-Trap confirmed that cMYC-StBSL1, cMYC-StBSL2, and cMYC-StBSL3 associated in *N. benthamiana* with GFP-tagged PiAVR2, but no association was seen with the GFP-PiAVR3a control. Expression of constructs in the leaves is indicated by +. Protein size markers are indicated in kilodaltons, and protein loading is indicated by Ponceau stain (PS).

The kelch-phosphatase StBSL1 was originally identified as a target of PiAVR2 in both potato and tomato (Saunders et al., 2012). A yeast two-hybrid (Y2H) library of complementary DNA (cDNA) made from potato infected with *P. infestans* (*Bos et al., 2010*) was screened with a GAL4 DNA binding domain-PiAVR2 fusion (“bait”) construct to a depth of 9 × 106 yeast cotransformants. Four independent yeast colonies recovered from selection plates that contained GAL4 activation domain (“prey”) fusions yielded sequences encoding StBSL3. In Arabidopsis, this family consists of four members: AtBSL1, AtBSL2, and AtBSL3, and BSU1. The bulk of research to date has focused on AtBSU1 with all four members linked to brassinosteroid pathway signaling (Mora-García et al., 2004; Kim et al., 2009, 2011). Basic Local Alignment Search Tool (BLAST) analysis of the *Solanum tuberosum* genome revealed only three family members in potato, with homologs of AtBSL1, AtBSL2, and AtBSL3 identified but no homolog of AtBSU1, which is regarded as Brassicaceae specific (Supplemental Fig. S1; Maselli et al., 2014). Y2H analysis revealed that not only full-length StBSL1, but also StBSL2 and StBSL3, interacted with the effector PiAVR2 while showing no interaction with the control effector Pi08949 (Fig. 1A). This was confirmed in planta using coimmunoprecipitation (co-IP), which again showed all three family members to specifically interact with PiAVR2, but not PiAVR3a (Fig. 1B), which was selected as a control as it shares a cytoplasmic localization with PiAVR2 in planta (Bos et al., 2010).

### Interaction with the BSL Family Is Essential for PIAVR2 Virulence Function

**Figure 2.**
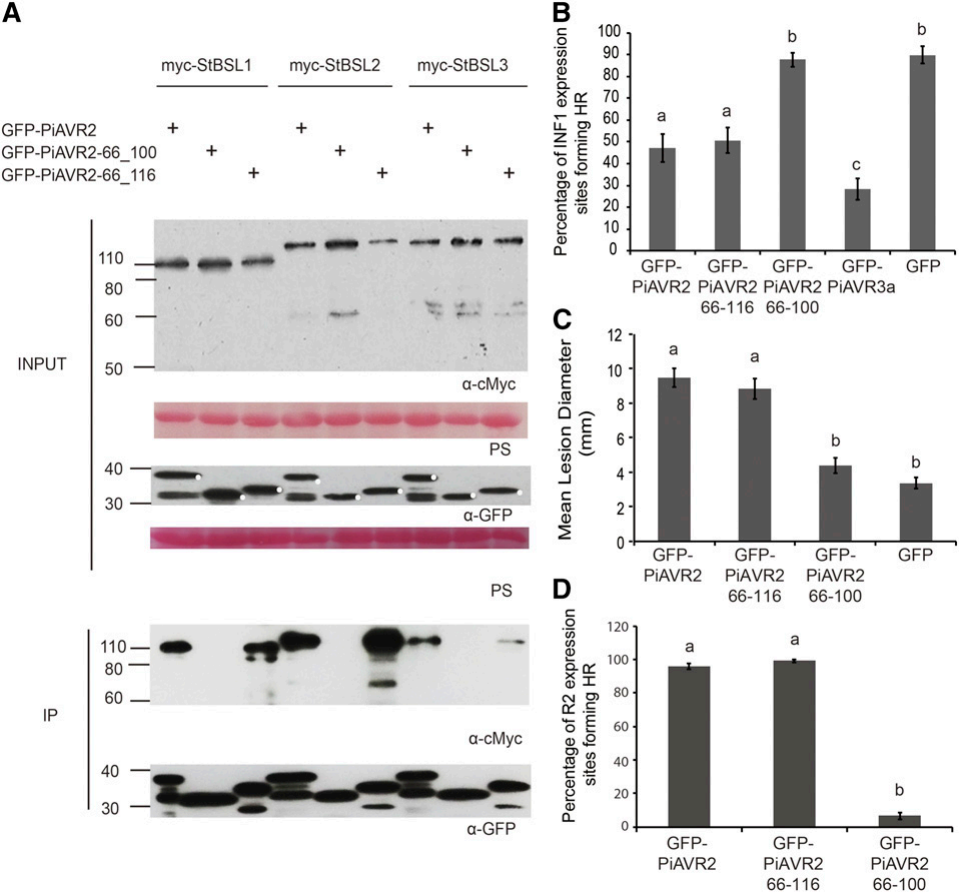
Interaction with the BSL family is essential for PiAVR2 virulence function. A, IP of protein extracts from agroinfiltrated leaves using GFP-Trap confirmed that cMYC-StBSL1, cMYC-StBSL2, and cMYC-StBSL3 associated in *N. benthamiana* with GFP-tagged PiAVR2 and PiAVR2-66_116, but no association was seen with GFP- PiAVR2-66_100. Expression of constructs in the leaves is indicated by +. Protein size markers are indicated in kilodaltons, and protein loading is indicated by Ponceau stain (PS). B, Transient coexpression of GFP-PiAVR2 or truncated forms of PiAVR2 with INF1 indicated that PiAVR2 and PiAVR2-66_116 can suppress ICD in *N. benthamiana,* similar to the GFP-AVR3a control, whereas PiAVR2-66_100 did not. Cell death sites were counted at 4 d post infiltration. Results combine data from three independent experimental replicates, each consisting of eight individual plants (biological replicates), with three leaves (technical replicates) infiltrated per plant. C, *P. infestans* lesion sizes at 8 d post inoculation of sporangia suspension (diameter in millimeters) following expression of GFP-tagged PiAVR2, PiAVR2-66_116, PiAVR2-66_100, and control GFP. Data shown combine 4 independent experimental replicates, each consisting of 18 leaves taken from 6 individual plants, with 4 inoculations per leaf. D, Transient coexpression of GFP-PiAVR2 or truncated forms of PiAVR2 with R2 indicated that PiAVR2 and PiAVR2-66_116 are recognized by R2 in *N. benthamiana*, whereas PiAVR2-66_100 is not. Cell death sites accounted at 3 d post infiltration. Results combine data from three independent experimental replicates, each consisting of 8 individual plants, with 3 leaves infiltrated per plant. Error bars indicate SEM. Significant difference in (B to D) is denoted by lowercase letters (*P* < 0.001 in one-way ANOVA, using the Student-Newman-Keuls method).

To identify a minimal region of PiAVR2 required for BSL interaction, a series of deletion constructs were generated (Supplemental Fig. S2) and GFP tagged for use in co-IP. Residues 1-65 were shown to be dispensable, with fragment 66-116 still able to facilitate interaction between PiAVR2 and all three BSLs (Fig. 2A). Further deletion of residues 101-116 to give PiAVR2 66-100 abolished PiAVR2-BSL interaction (Fig. 2A), suggesting that this 16-amino acid region is essential for binding. It may contain the site of binding itself or potentially be a critical region for maintaining the required protein conformation necessary for AVR2-BSL interaction.

Many effectors play a role in pathogenicity by means of immune suppression. Recognition of the oomycete elicitin INF1, leading to localized cell death, can be used as a readout for successful immune signaling (Kamoun et al., 1997; Du et al., 2015). PiAVR2 has been shown previously to suppress INF1-triggered cell death (ICD; Turnbull et al., 2017), as do several other characterized *P. infestans* effectors, such as PiAVR3a, Pi18215, Pi02860, and Pi17316 (*Bos et al., 2010*; Zheng et al., 2014; Yang et al., 2016; He et al., 2018b; Murphy et al., 2018). Transient coexpression of INF1 with full-length and truncated forms of PiAVR2 was used to assess the extent of immune suppression. PiAVR2 66-116 was shown to suppress ICD to the same extent as the full-length effector (Fig. 2B). However, removal of residues 100-116 rendered PiAVR2 unable to suppress ICD (Fig. 2B). Crucially, this also translates into pathogenicity, with transient expression of PiAVR2 66-100 providing no enhancement of *P. infestans* colonization on *Nicotiana benthamiana* (Fig. 2C), whereas PiAVR2 66-116 expression facilitated a significant increase in lesion size, comparable to the full-length effector.

StBSL1 has been shown to be required for PiAVR2 recognition by the NB-LRR protein R2, with *BSL1* silencing in *N. benthamiana* resulting in a significant decrease in R2-mediated HR (Saunders et al., 2012). Figure 2D supports a model in which PiAVR2-BSL interaction is required for R2 activation. PiAVR2 66-116, which maintains BSL interaction, is recognized to the same extent as the full-length effector triggering a full HR. In contrast, PiAVR2 66-100 is unable to trigger R2-mediated HR, suggesting that impaired BSL interaction has abolished effector recognition.

### *BSL*-Silenced Plants Show Enhanced Immune Responses

Virus-induced gene silencing (VIGS) constructs were designed to transiently reduce expression of the *BSL* genes in *N. benthamiana* (Saunders et al., 2012; this study). High sequence similarity prevented the design of *NbBSL2* and *NbBSL3*-specific silencing constructs, so dual-silencing constructs were generated to reduce both *NbBSL2* and *NbBSL3* expression simultaneously. *NbBSL1*-silenced plants appeared phenotypically normal, whereas *NbBSL2/3*-silenced plants exhibited severe dwarfism with short petioles and curled, brittle leaves (Supplemental Fig. S3), reminiscent of *AtBRI1* knockout mutants in the literature (Clouse et al., 1996; Noguchi et al., 1999). Reverse transcription quantitative PCR (RT-qPCR) confirmed effective silencing of the *NbBSLs* using these constructs and revealed a striking relationship between expression levels of *NbBSL1* and *NbBSL2/3*. When *NbBSL1* expression is silenced, *NbBSL2* and *NbBSL3* transcripts increase between 1.5- and 2-fold. Similarly, when *NbBSL2* and *NbBSL3* are simultaneously silenced, *NbBSL1* transcripts accumulate almost 3-fold (Supplemental Fig. S3). This may be a compensatory or feedback mechanism to attempt to achieve homeostasis of BR signaling in the absence of one or multiple members. However, immunoblot analysis of all three StBSLs in the *NbBSL*-silenced plants revealed that an increased accumulation of *BSL1* transcripts may not necessarily translate into increased protein level. StBSL2 and StBSL3 are undetectable in *BSL2/3*-silenced plants but remain detectable in *BSL1*-silenced plants, as expected. Also as anticipated, StBSL1 is undetectable in *BSL1*-silenced plants. However, unexpectedly, StBSL1 protein was undetectable in *NbBSL2/3*-silenced plants as well (Supplemental Fig. S4). This suggests a regulatory relationship between the BSLs at the protein level; it may be that BSL2, BSL3, or both are required for BSL1 stability, whether directly or indirectly. The *NbBSL2/3* silencing construct effectively creates a BSL-null plant. VIGS construct sequences can be seen in Supplemental Figure S5.

**Figure 3.**
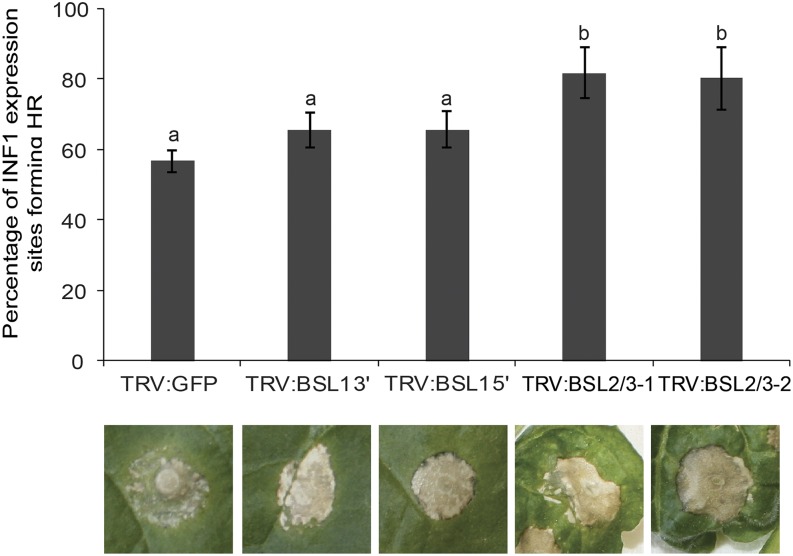
Silencing of the *BSL* family increases INF1-triggered cell death in *Nicotiana benthamiana*. Silencing *BSL1* slightly increases ICD, and *BSL2/3* significantly accelerates ICD at 4 d post infiltration of *Agrobacterium* allowing transient expression of INF1. Silencing was achieved using VIGS. Significant difference is denoted by lowercase letters (*P* < 0.001 in one-way ANOVA, using the Student-Newman-Keuls method). Results shown are a combination of data from three independent experimental replicates, each consisting of seven individual plants, with three leaves infiltrated per plant. Error bars indicate SEM. Representative ICD lesions are shown.

**Figure 4.**
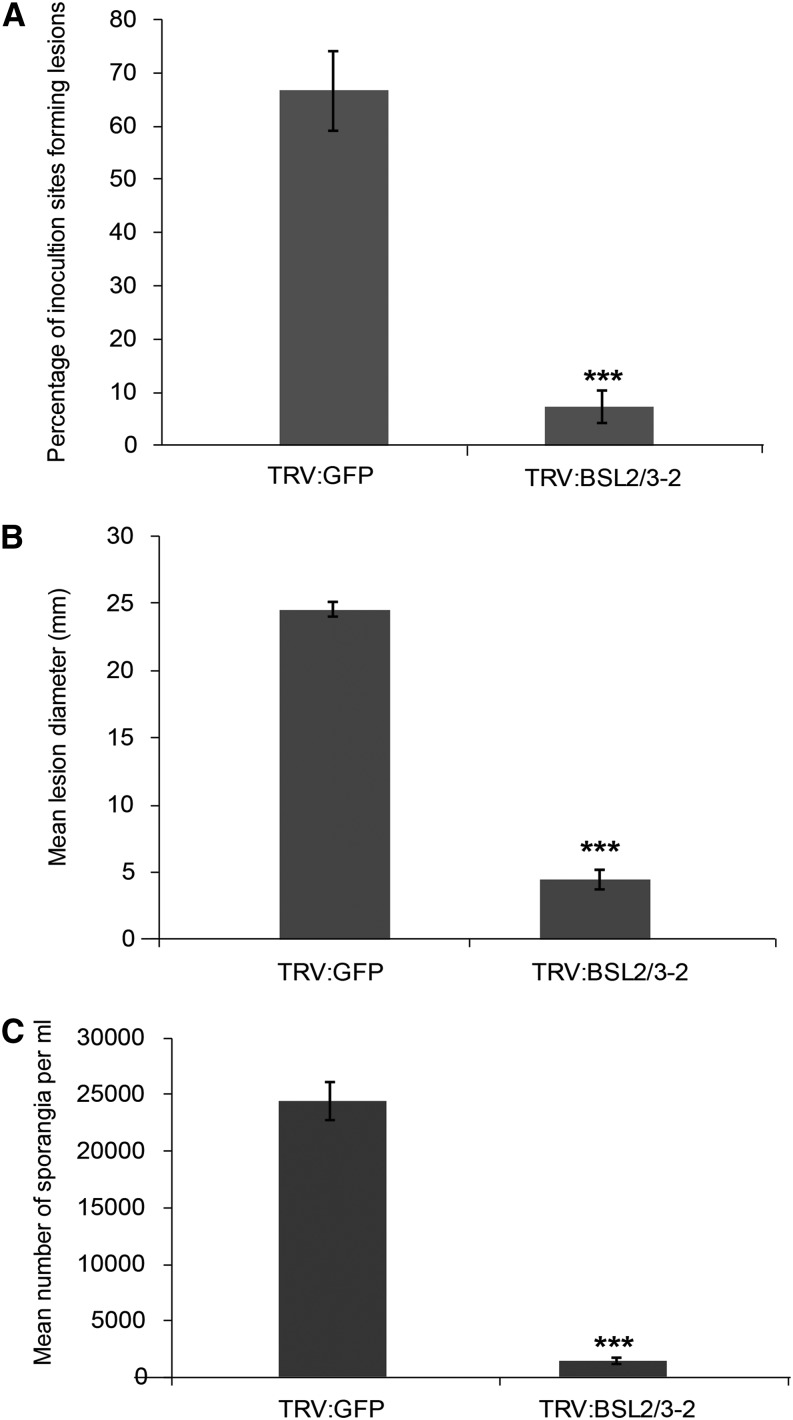
VIGS of *BSL2* and *BSL3* in *Nicotiana benthamiana* resulted in decreased susceptibility to *P. infestans. BSL2/3-*silenced plants were inoculated with a *P. infestans* zoospore suspension and a variety of measurements made at 7 d post inoculation: percentage of inoculation sites forming lesions (A), lesion diameter (B), and number of sporangia per milliliter (C). Results were combined from four independent experimental replicates, each involving 8 individual plants, with three leaves inoculated per plant. Error bars indicate SEM. Significant difference is represented by asteriks (***P < 0.01 in one way ANOVA, using the Student-Newman-Keuls method).

*NbBSL*-silenced plants were screened for ICD as a readout for strength of immune response. Silencing *NbBSL1* resulted in a trend toward an increase in ICD, although this did not reach statistical significance. However, a pronounced increase in ICD was observed in *NbBSL2/3*-silenced plants (Fig. 3). This suggests that negative regulation of PTI has been removed or reduced, allowing a stronger response to PAMP recognition. This increased immune response might be expected to hinder *P. infestans* infection. Previous work showed *NbBSL1* silencing alone to have no clear effect on pathogen virulence (Saunders et al., 2012). To investigate whether *NbBSL2/3* silencing, effectively creating a BSL-null scenario, impacts *P. infestans* pathogenicity, *NbBSL2/3-*silenced plants were inoculated with zoospore suspension and a variety of infection parameters measured. Percentage of inoculation sites forming lesions, lesion diameter, and number of sporangia recovered all show a striking decrease in successful infection in the absence of the BSLs (Fig. 4).

### BSL1, BSL2, and BSL3 Are Susceptibility Factors

**Figure 5.**
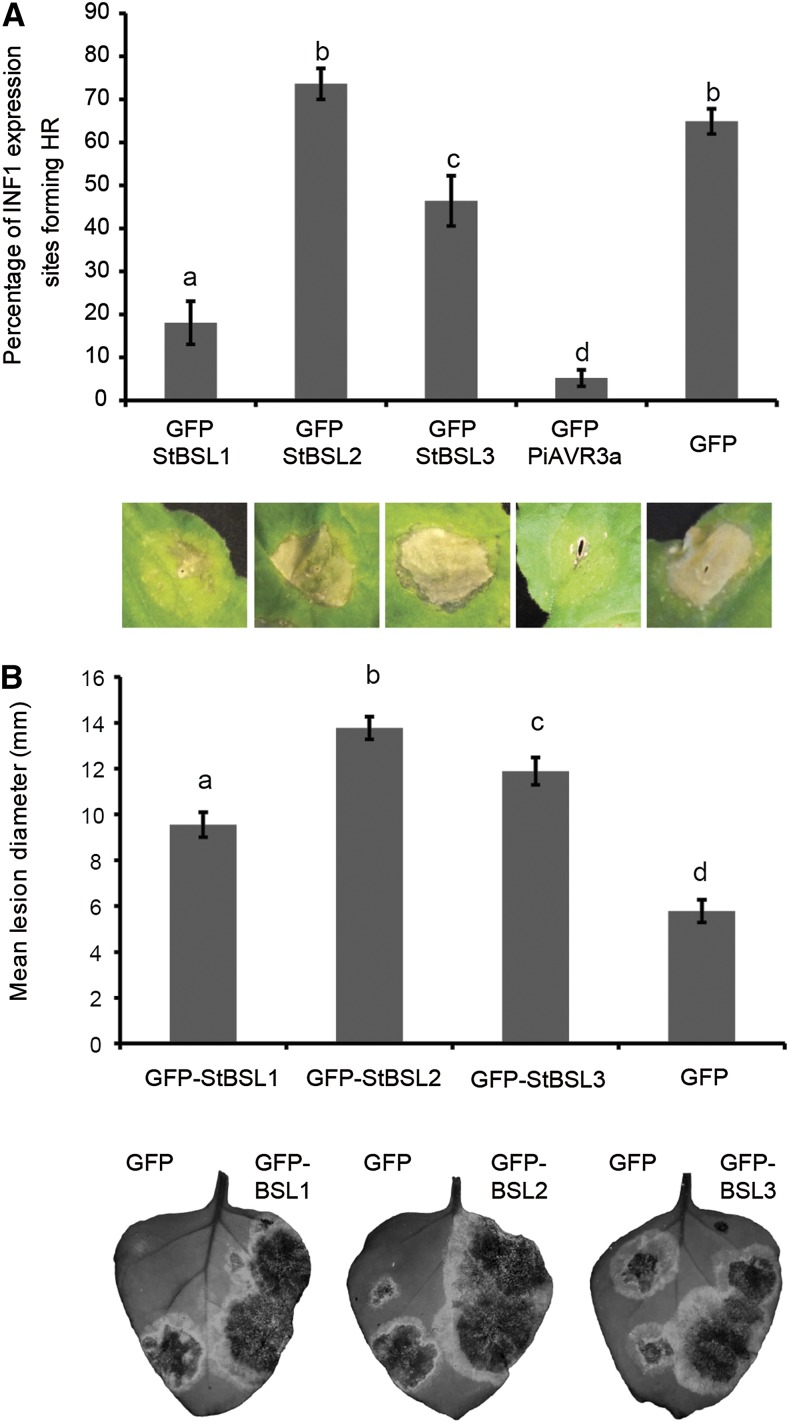
The BSL family suppresses immunity and enhances *P. infestans* leaf colonization. A, INF1-triggered cell death following coexpression of INF1 with GFP-StBSL1, GFP-StBSL2, and GFP-StBSL3 transiently expressed in *N. benthamiana*. Data are a combination of 3 independent experimental replicates, consisting of eight individual plants, with three leaves infiltrated per plant. Representative ICD lesions are shown. B, *P. infestans* lesion sizes following transient expression of GFP-StBSL1, GFP-StBSL2, and GFP-StBSL3 in *N. benthamiana* at 8 d post inoculation of sporangia. Data are a combination of 3 independent experimental replicates, each involving 15 leaves from 7 individual plants. Significant differences in (A) and (B) are represented by lowercase letters (*P* < 0.05 in one-way ANOVA, using the Student-Newman-Keuls method). Error bars indicate SEM. Representative leaf images show the full extent of the lesion under UV light, converted to grayscale.

Pathogen effectors are often responsible for inhibiting the function of target proteins (e.g. King et al., 2014). Previously, we have shown that silencing NbBSL1 had no discernable effect on *P. infestans* lesion development (Saunders et al., 2012). Here, we show that silencing NbBSL2 and NbBSL3 in combination (indirectly also reducing BSL1 protein level) significantly reduced infection. This suggests that *P. infestans* would not benefit from negatively affecting BSL function. On the contrary, the pathogen may benefit from increased BSL levels or activity. To determine whether overexpression of the StBSLs has an effect on the plant immune response, we first examined ICD following coexpression of INF1 with free GFP, with GFP-tagged StBSL1/2/3, or with GFP-tagged PiAVR3a as a known suppressor of ICD. Scoring the percentage of ICD reveals StBSL1 to have a strong suppressive effect, StBSL3 to have a moderate effect, and BSL2 to have no effect (Fig. 5A). To determine the effect of BSL overexpression on *P. infestans* infection, StBSL1, StBSL2, or StBSL3 was transiently expressed in one half of an *N. benthamiana* leaf, with free GFP expressed in the other half, and the leaf was inoculated 1 d later on both sides with zoospore suspension. In each case, expression of StBSL1, StBSL2, or StBSL3 resulted in a significant increase *in P. infestans* infection (Fig. 5B). Together, the VIGS and overexpression studies demonstrate the status of the BSL family as “S factors,” plant proteins that the pathogen requires to reach full infection potential.

**Figure 6.**
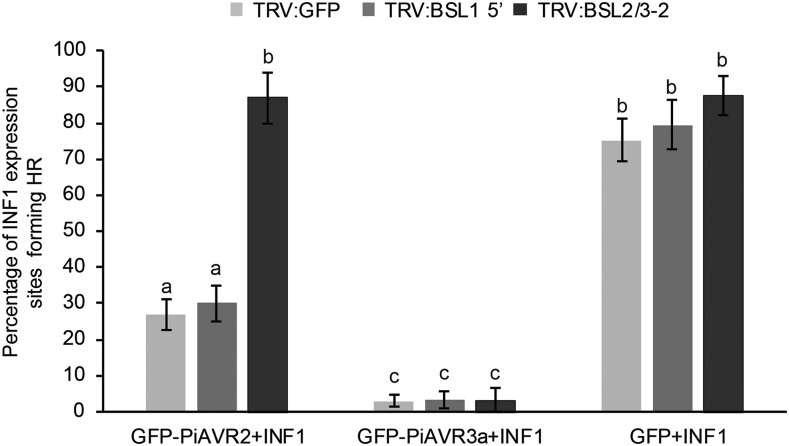
Silencing of *BSL2/3* compromises the suppression of INF1-triggered cell death by PiAVR2 in *Nicotiana benthamiana*. Coinfiltration INF1 with GFP-PiAVR2, GFP-PiAVR3a, and GFP control in TRV:BSL1, TRV:BSL2/3, and TRV:GFP plants. Cell death sites were counted at 5 d post infiltration. Significant difference was represented by letters (*P* < 0.001 in one-way ANOVA, using the Student-Newman-Keuls method). Results are combined data across three independent experimental replicates, consisting of seven individual plants, with three leaves infiltrated per plant. Error bars indicate SEM.

The ability of PiAVR2 to suppress ICD was further characterized using VIGS of NbBSL1 or NbBSL2/3. While PiAVR3a remains able to suppress ICD regardless of NbBSL1 or NbBSL2/3 silencing, PiAVR2 could suppress ICD only in the NbBSL1-silenced plants and not in the NbBSL2/3-silenced plants, which are effectively BSL null (Fig. 6).

### StBSL Immune Suppression Requires the Downstream Susceptibility Factor CHL1

**Figure 7.**
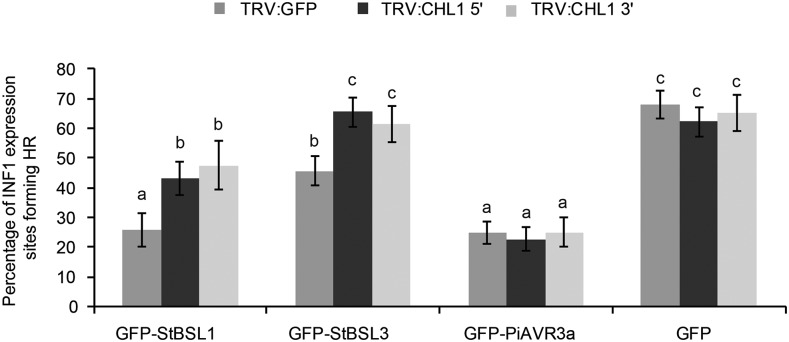
Silencing of *CHL1* attenuated the suppression of INF1-triggered cell death by GFP-BSL1 and GFP-BSL3 in *Nicotiana benthamiana*. INF1 was coinfiltrated with GFP-StBSL1, GFP-StBSL3, GFP-AVR3a, and GFP control in TRV:CHL1 and TRV:GFP control plants. Cell death sites were counted at 5 d post infiltration. Significant difference is represented by letters (*P* < 0.001 in one-way ANOVA, using the Student-Newman-Keuls method). Results shown are combined data across three independent biological replicates, each consisting of seven individual plants, with three leaves infiltrated per plant. Error bars indicate SEM.

Work in our group previously identified the transcription factor StCHL1 as a negative regulator of immunity in *N. benthamiana* and potato (Turnbull et al., 2017). CHL1 was shown to suppress ICD and to increase *P. infestans* leaf infection. Moreover, the ability of PiAVR2 to suppress ICD was dependent on CHL1. To determine whether ICD suppression by BSL1 and BSL3 is also CHL1 dependent, *CHL1* was transiently silenced in *N. benthamiana*, followed by coexpression of INF1 plus StBSL1, StBSL3, PiAVR3a, or free GFP as a control. ICD was suppressed by PiAVR3a independent of *NbCHL1* silencing. In contrast, StBSL1 and StBSL3 showed a significant decrease in ICD suppression on *NbCHL1*-silenced plants. While StBSL1 was still able to achieve moderate, albeit significantly reduced, suppression in the *CHL1*-silenced plants, StBSL3 was unable to suppress ICD at all (Fig. 7).

## DISCUSSION

**Figure 8.**
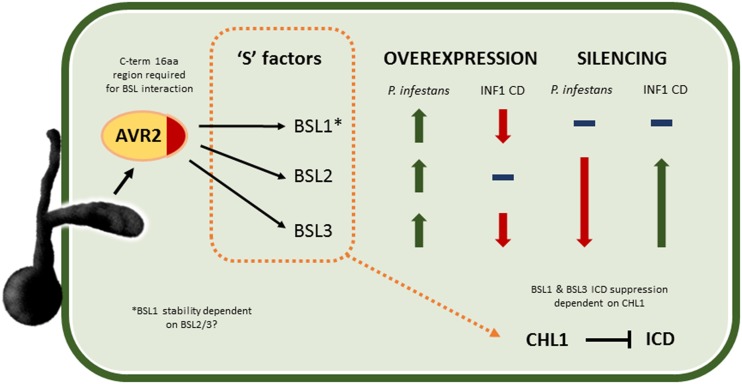
Schematic diagram illustrating main findings of this work. AVR2 is an RXLR effector secreted into the plant by *P. infestans,* where it interacts with the kelch-repeat phosphatases StBSL1, 2, and 3. A 16-amino acid region at the C terminus (C-term) of the effector is shown to be essential for BSL interaction. All three BSL family members can be considered to be susceptibility S factors, with overexpression increasing *P. infestans* leaf infection. Overexpression of StBSL1 and BSL3 can suppress ICD, with no effect seen for StBSL2. Silencing StBSL1 results in no change to *P. infestans* leaf infection or to ICD. Silencing StBSL2 and 3 in combination significantly reduces *P. infestans* leaf infection and significantly increases ICD. Notably, silencing StBSL2 and 3 reduces protein level of StBSL1, suggesting that one or both of these are required for StBSL1 stability. Finally, suppression of ICD by StBSL1 and StBSL3 has been shown to require the transcription factor StCHL1, a suppressor of immunity in Solanaceous plants.

In this work we have determined that the *P. infestans* effector PiAVR2 targets all three members of the BSL family in *S. tuberosum*: StBSL1, StBSL2, and StBSL3 (Fig. 1). Transient overexpression of each is able to enhance leaf colonization by *P. infestans*, identifying these proteins as susceptibility (S) factors in late blight infection (Fig. 5B). This family of kelch-repeat phosphatases is homologous to Arabidopsis BSU1, characterized as a positive regulator in the growth-promoting brassinosteroid signaling pathway. Previous work had established that PiAVR2 interacts with StBSL1 (Saunders et al., 2012) and that PiAVR2 upregulates brassinosteroid-induced genes, exploiting the crosstalk that exists between growth and immunity in plants (Turnbull et al., 2017). The main findings of this work are represented in Figure 8.

We identified that interaction between PiAVR2 and the StBSL family requires a specific C-terminal region of the effector (Fig. 2A). Crucially, loss of this region, and, thus, loss of BSL interaction, strips PiAVR2 of its virulence function. The truncated effector PiAVR2 66-100 can no longer suppress ICD and can no longer enhance *P. infestans* leaf colonization (Fig. 2, B and C). This region of interest contains sequence similarity to known phosphatase interaction motifs. KKLV (PiAVR2 102-105) is reminiscent of the KKVI of BSU1, recently shown to be required for oligomerization between BSL family members in Arabidopsis (Kim et al., 2016). Another, LKIKG (PiAVR2 108-112), contains the same residues as the PROTEIN PHOSPHATASE 1 (PP1) binding sequence KGILK (also referred to as the GILK, or SILK, motif), essential for potent inhibition of PP1 by the inhibitor I2 (Huang et al., 1999; Connor et al., 2000). Phosphatases, in contrast to kinases, are relatively few in number in the plant proteome. Where kinases have increased specificity by means of gene duplication and specialization, phosphatases have achieved diversity by interacting with a large number of regulatory subunits, forming “holoenzymes” (Hendrickx et al., 2009). These regulatory subunits can specify substrate or localization, act as inhibitors or chaperones, or a combination of these roles (Wakula et al., 2003). This raises the possibility that PiAVR2 acts to modify oligomerization between the BSLs, by occluding an interaction site, or perhaps favoring a particular BSL combination. Alternatively, or as a result of this, it may act to enhance or inhibit BSL activity on a particular substrate. Modification of plant phosphatase function by pathogenic effectors is not unprecedented; the *P. infestans* effector Pi04314 has recently been shown to interact with three PP1c isoforms in potato, resulting in their relocalization from the nucleolus to the nucleoplasm, with PP1c activity required for successful infection (Boevink et al., 2016a). Detailed examination of BSL oligomerization in *S. tuberosum*, identification of substrates, and analysis of enzymatic activity in the presence/absence of PiAVR2 will be an important next step in elucidating effector function.

While all three StBSL family members interact with PiAVR2, and all enhance pathogen virulence when overexpressed, this is not a straightforward case of functional redundancy. There are several key differences between the BSL family members, which show functional differences as well as regulatory interactions between family members at both the gene expression and protein levels. Particularly striking is the loss of StBSL1 protein in *NbBSL2/3*-silenced plants, suggesting that BSL2 or 3 is required to maintain BSL1 stability (Supplemental Fig. S4). StBSL2 also presents an interesting discrepancy, showing no ICD suppression while still able to provide a significant enhancement to *P. infestans* infection (Fig. 5). This implies that BSL2 mode of action may be distinct from ICD suppression or that PiAVR2 is not simply increasing the normal activity of StBSL2 but may be modifying it to facilitate ICD suppression.

We recently showed that PiAVR2 requires the solanaceous transcription factor CHL1 for full virulence function (Turnbull et al., 2017). *CHL1* expression is upregulated by brassinosteroid signaling and functions as a negative regulator of plant innate immunity. We revisited *NbCHL1* silencing to determine whether ICD suppression by StBSL1 and StBSL3 also requires this transcription factor; while StBSL1 retained some limited ability to suppress cell death, StBSL3 was rendered unable to do so on *NbCHL1* VIGS plants (Fig. 7). This again highlights differences between the BSLs and may reflect interdependency between family members. If the BSLs function together, as is suggested by the report of oligomerization (Kim et al., 2016), then one protein may require higher levels of the other family members to take full effect.

In conclusion, this work identifies the StBSL family as targets of *P. infestans* PiAVR2, the first filamentous pathogen effector shown to exploit crosstalk between the brassinosteroid pathway and immune signaling in plants. All three phosphatases act as S factors in *P. infestans* infection, allowing increased disease potential when overexpressed and decreased disease when silenced in combination. The next step will be to elucidate the biochemical mechanism behind PiAVR2-mediated immune suppression and that of R2 recognition. Is BSL phosphatase activity required for immune suppression or PiAVR2 recognition? How does PiAVR2 affect oligomerization and downstream activity/substrate specificity of the BSL family? This work also raises interesting areas for inquiry with regard to the BSL family members, which do not act with the functional redundancy once thought. Each member of the brassinosteroid signaling cascade is a member of a multigene family, and while redundancy does exist to an extent, the true picture is likely to be more complex with family members potentially playing distinct roles.

S factors such as the BSLs present an interesting avenue to explore in terms of breeding for disease resistance. While R protein-mediated resistance is highly effective, it is also highly specific, with success often short-lived due to rapidly evolving pathogen populations. Subtle manipulation of the balance between growth and immunity may be a more indirect way of providing plant protection. Identification of S factors provides insight not only into disease processes, but also into the fundamentals of healthy plant function, knowledge that can be exploited in our ongoing quest for disease resistance and sustainable food security.

## MATERIALS AND METHODS

### Plant Material

*Nicotiana benthamiana* plants were grown in general purpose compost under long-day glasshouse conditions of 16-h light at 22°C, light intensity of 130 to 150 µE m^−2^ s^−1^, and 40% humidity. Plants were used for cell death*/Phytophthora infestans* colonization at 4 to 5 weeks old.

### Constructs and Cloning

StBSL1, 2, and 3 were amplified from *Solanum tuberosum* cDNA using gene-specific primers (Eurofins) modified with attB Gateway recombination sites (Invitrogen), before recombination into pDONR201 to generate entry clones by BP reaction (BP clonase, Invitrogen). LR clonase (Invitrogen) was used to recombine genes of interest into pB7WGF2 (N-terminal GFP tag) or pGWB18 (N-terminal myc tag). Primer sequences can be found in Supplemental Table S1.

INF1, GFP-PiAVR2, and GFP-PiAVR3a constructs were generated as previously described (Gilroy et al., 2011b; Engelhardt et al., 2012).

### *Agrobacterium*-Mediated Transient Expression and Cell Death Assays

*Agrobacterium* strain AGL1 VirG pSOUP was transformed with the constructs of interest by electroporation and screened by colony PCR. Liquid YEB medium was inoculated with single colonies from plates containing selective antibiotics and incubated with shaking overnight at 28°C. Bacteria were pelleted by centrifugation at 4,000*g* for 10 min, with the pellet resuspended in 10 mm MES and 10 mm MgCl. OD_600_ was adjusted as appropriate, and acetosyringone added to 200 µM. Leaves were infiltrated on the abaxial surface, using a 1-mL syringe after needle wounding.

For co-IPn, western blotting, and cell death assays, cultures were infiltrated at an OD_600_ of 0.5, with an OD_600_ of 0.1 used for *P. infestans* leaf colonization assays. *Agrobacterium* with the silencing suppressor construct pJL3-P19 was coinfiltrated at an OD_600_ of 0.05.

Hypersensitive response/INF1-triggered cell death assays were performed by coinfiltration of the relevant constructs (infiltration site ∼1 cm diameter) and scoring sites positive/negative for cell death after 7 d. A positive score was determined as 50% or more of the infiltrated area showing cell death. Independent experimental replicates consisted of 6 or more individual plants (biological replicates), with multiple leaves (technical replicates) infiltrated per plant. Data from each biological replicate were used to perform statistical analysis using one-way ANOVA (Newman-Keuls method) in Sigmaplot (Systat Software).

### Virus-Induced Gene Silencing

VIGS constructs consisted of approximately 250-bp PCR fragments of the gene targeted for silencing, cloned into pBinary Tobacco Rattle Virus (TRV) vectors (Liu et al., 2002). A TRV construct expressing a fragment of GFP was used as a control (Gilroy et al., 2011a), and BSL1 and CHL1 constructs were as previously described (Saunders et al., 2012; Turnbull et al., 2017). *BSL2/3* silencing fragments were amplified from *N. benthamiana* cDNA using the primers BSL2/3-1 F and BSL2/3-1 R, and BSL2/3-2 F and BSL2/3-2 R (see Supplemental Table S1). To achieve transient silencing, the two largest leaves of *N. benthamiana* plants at the four-leaf stage were fully syringe infiltrated with a mixture of *Agrobacterium* strain LBA4404 containing TRV-RNA1 and the silencing fragment of interest, each at an OD_600_ of 0.25. Viral infection was allowed to progress systemically for 2 to 3 weeks before the plants were used in experiments.

### RT-qPCR

RNA was extracted from plant tissue using the Qiagen RNeasy Plant Mini Kit, complete with on-column DNase treatment, and used to synthesize cDNA with SuperScript II reverse transcriptase (Invitrogen) both according to the manufacturers’ instructions. RT-qPCR was performed using Maxima SYBR green qPCR mastermix (Thermo Fisher Scientific) with detection using a Chromo4 real-time detector with an MJ Research PTC-200 thermal cycler and Opticon Monitor v. 3.1.32 software (Bio-RAD Laboratories). Reactions were incubated at 95°C for 15 min, followed by 40 cycles (95°C for 15 s, 60°C for 1 min, plate read, hold 5 s). Data were analyzed using the ΔΔCt method (Schmittgen and Livak, 2008), with expression normalized to the housekeeping gene *NbEF1α*. Primers were generated by Eurofins MWG Operon, with primer design facilitated by NetPrimer software (PREMIER Biosoft; http://www.premierbiosoft.com/netprimer/; Supplemental Table S1).

### Y2H Assays

Y2H screening was carried out using the ProQuest system (Invitrogen). PiAVR2 and the control effector Pi08949 were ligated into the prey vector pDEST22, with StBSL1, StBSL2, and StBSL3 ligated into the bait vector pDEST32. Pairwise interaction was tested using bait and prey to transform the yeast strain MaV203. Transformants were identified by selection on media lacking Leu and Trp. These were screened for HIS3 induction and URA3 induction by plating on the appropriate dropout media and for β-galactosidase induction by 5-bromo-4-chloro-3-indolyl-β-d-galactopyranoside acid assay according to the manufacturer’s instructions.

### Western blot

To examine protein levels, *N. benthamiana* was infiltrated with the relevant *Agrobacterium* transformants, with leaf tissue sampled 48 h postinfiltration and immediately frozen in liquid nitrogen. Protein extraction was performed by heating ground tissue samples at 95°C for 10 min in 2x SDS gel-loading buffer (100 nm Tris-HCl, pH 6.8, 0.2% [w/v] bromophenol blue, 20% [v/v] glycerol, and 4% [w/v] SDS), followed by centrifugation at 16,000*g* for 5 min. Proteins were separated on 4% to 12% Bis-Tris PAGE gels with MES buffer, using an X-blot Mini Cell (Thermo Fisher Scientific), followed by transfer to nitrocellulose membrane (GE Healthcare Life Sciences) using an X10 Blot Module (Thermo Fisher Scientific) according to the manufacturer’s instructions. Membranes were stained with Ponceau solution to confirm transfer and even loading.

Membranes were blocked in 4% milk in 1x PBS 0.1% Tween (1xPBS-T) with shaking overnight at 4°C, before incubation with the appropriate antibodies (Santa Cruz sc-40 and sc-9996).

Signal was visualized by incubation with Amersham ECL Prime, on Amersham ECL Hyperfilm (both GE Healthcare Life Sciences), developed with a Compact X4 Automatic Processor (Xograph Healthcare Ltd).

### Co-IP

For co-IP, proteins were extracted in GTEN buffer (10% [v/v] glycerol, 25 mm Tris-HCl [pH 7.5], 1 mm EDTA, and 150 mm NaCl) with 10 mm dithiothreitol, protease inhibitor cocktail, 1 mm phenylmethylsulfonyl fluoride, and 0.2% Nonidet P-40. To immunoprecipitate GFP-tagged proteins, protein extracts were incubated with GFP-Trap_M beads (Chromotek) for 1 h at 4°C. Beads were washed three times in GTEN buffer with phenylmethylsulfonyl fluoride and protease inhibitor cocktail as above, before resuspending in 2x SDS gel-loading buffer. Samples were then processed by western blot as described above.

### *P. infestans* colonization assays

*P. infestans* strain 88069 expressing fluorescent protein tdTomato (Saunders et al., 2012; McLellan et al., 2013) was grown on rye agar supplemented with 20 µg/mL geneticin. Ten-day-old plates were flooded with sterile distilled water and sporangia harvested using a 70-µm cell strainer. The suspension was centrifuged at 3,000*g* for 10 min, supernatant discarded, and pellet resuspended in sterile distilled water. Sporangia were quantified using a hemocytometer and adjusted to 50,000 per mL^−1^. Droplets (10 µL) were pipetted onto the abaxial surface of detached leaves, placed in sealed boxes with moist tissue to maintain humidity, and kept in darkness for 24 h to reduce UV degradation of inoculum. Lesions were measured at the widest point 7 d post inoculation.

If required, leaves were infiltrated with appropriate agrobacterium constructs 24 h presporangia inoculation to achieve transient expression of the gene of interest.

Data from infection and cell death assays were subjected to statistical analysis using one-way ANOVA (Newman-Keuls method) in Sigmaplot (Systat Software)

### Accession Numbers

Sequence data from this article can be found in the GenBank/EMBL data libraries under accession numbers KY852490.1, XM_002902939.1, and NM_001318640.1.

### Supplemental Data

The following supplemental materials are available:

**Supplemental Figure S1.** Phylogenetic tree of BSL family members from potato and Arabidopsis.
**Supplemental Figure S2.** Protein alignment of PiAVR2 full length, PiAVR2 66-116 and PiAVR2 66-100.
**Supplemental Figure S3.** *NbBSL* expression levels and phenotypes of *NbBSL1*, *NbBSL2* and *NbBSL3* silenced *N. benthamiana*.
**Supplemental Figure S4.** Protein stability of 35S promoter-driven GFP-BSL1, GFP-BSL2 and GFP-BSL3 transiently expressed in VIGS N. benthamiana plants expressing TRV:BSL1 or TRV:BSL2/3 constructs as indicated.
**Supplemental Figure S5.** Nucleotide alignment of the *N. benthamiana* BSL sequences cloned in TRV constructs with the same region of potato BSL1, 2 and 3.
**Supplemental Table S1.** Primers used in the study.
